# Assessment of Response to Neoadjuvant Systemic Treatment in Triple-Negative Breast Cancer Using Functional Tumor Volumes from Longitudinal Dynamic Contrast-Enhanced MRI

**DOI:** 10.3390/cancers15041025

**Published:** 2023-02-06

**Authors:** Bikash Panthi, Beatriz E. Adrada, Rosalind P. Candelaria, Mary S. Guirguis, Clinton Yam, Medine Boge, Huiqin Chen, Kelly K. Hunt, Lei Huo, Ken-Pin Hwang, Anil Korkut, Deanna L. Lane, Huong C. Le-Petross, Jessica W. T. Leung, Jennifer K. Litton, Rania M. Mohamed, Benjamin C. Musall, Sanaz Pashapoor, Miral M. Patel, Frances Perez, Jong Bum Son, Alastair Thompson, Vicente Valero, Peng Wei, Jason White, Zhan Xu, Lawrence Pinsky, Debu Tripathy, Wei Yang, Jingfei Ma, Gaiane M. Rauch

**Affiliations:** 1 Department of Imaging Physics, The University of Texas MD Anderson Cancer Center, Houston, TX 77030, USA; 2 Department of Physics, University of Houston, Houston, TX 77030, USA; 3 Department of Breast Imaging, The University of Texas MD Anderson Cancer Center, Houston, TX 77030, USA; 4 Department of Breast Medical Oncology, The University of Texas MD Anderson Cancer Center, Houston, TX 77030, USA; 5 Department of Biostatistics, The University of Texas MD Anderson Cancer Center, Houston, TX 77030, USA; 6 Department of Breast Surgical Oncology, The University of Texas MD Anderson Cancer Center, Houston, TX 77030, USA; 7 Department of Pathology, The University of Texas MD Anderson Cancer Center, Houston, TX 77030, USA; 8 Department of Bioinformatics and Computational Biology, The University of Texas MD Anderson Cancer Center, Houston, TX 77030, USA; 9 Department of Surgery, Baylor College of Medicine, Houston, TX 77030, USA; 10 Department of Abdominal Imaging, The University of Texas MD Anderson Cancer Center, Houston, TX 77030, USA

**Keywords:** neoadjuvant systemic therapy, response prediction, triple-negative breast cancer, functional tumor volume, breast MRI

## Abstract

**Simple Summary:**

Neoadjuvant systemic therapy (NAST) is given before surgery to reduce tumor burden in patients with triple-negative breast cancer (TNBC), which is an aggressive breast cancer subtype that accounts for approximately 30% of breast cancer-related mortalities. Unfortunately, approximately 50% of TNBC patients do not respond to NAST and develop distant spread within 5 years. Reliable clinical methods are needed to determine non-responders to NAST in order to avoid the severe toxicity of ineffective regimens and offer novel targeted treatments. The purpose of this study was to investigate functional tumor volume measured from dynamic contrast-enhanced MRI for early assessment of NAST response in TNBC. Our study demonstrated the potential of functional tumor volume, evaluated as early as after 2 and 4 cycles of NAST, to serve as a non-invasive biomarker for the prediction of treatment response in TNBC patients.

**Abstract:**

Early assessment of neoadjuvant systemic therapy (NAST) response for triple-negative breast cancer (TNBC) is critical for patient care in order to avoid the unnecessary toxicity of an ineffective treatment. We assessed functional tumor volumes (FTVs) from dynamic contrast-enhanced (DCE) MRI after 2 cycles (C2) and 4 cycles (C4) of NAST as predictors of response in TNBC. A group of 100 patients with stage I-III TNBC who underwent DCE MRI at baseline, C2, and C4 were included in this study. Tumors were segmented on DCE images of 1 min and 2.5 min post-injection. FTVs were measured using the optimized percentage enhancement (PE) and signal enhancement ratio (SER) thresholds. The Mann–Whitney test was used to compare the performance of the FTVs at C2 and C4. Of the 100 patients, 49 (49%) had a pathologic complete response (pCR) and 51 (51%) had a non-pCR. The maximum area under the receiving operating characteristic curve (AUC) for predicting the treatment response was 0.84 (*p* < 0.001) for FTV at C4 followed by FTV at C2 (AUC = 0.82, *p* < 0.001). The FTV measured at baseline was not able to discriminate pCR from non-pCR. FTVs measured on DCE MRI at C2, as well as at C4, of NAST can potentially predict pCR and non-pCR in TNBC patients.

## 1. Introduction

Triple-negative breast cancer (TNBC) is a subtype of breast cancer that lacks expression of estrogen receptor, progesterone receptor, and human epidermal growth factor receptor 2 (HER2). TNBC accounts for approximately 10% to 20% of all breast cancers but approximately 30% of breast cancer-related mortalities [1]. Endocrine therapy, commonly used to treat estrogen receptor–positive and progesterone receptor–positive breast cancers, and HER2-targeted therapies, used to treat HER2-positive breast cancers, are ineffective against TNBC. Neoadjuvant systemic therapy (NAST) followed by surgery is a standard treatment strategy for TNBC despite high toxicity [2]. Approximately 50% of patients with TNBC who receive NAST achieve a pathologic complete response (pCR), defined as no residual invasive disease in the surgical specimen. Patients with a pCR have a good prognosis with respect to long-term survival and other clinical outcomes. Unfortunately, patients who do not achieve a pCR after NAST have substantially higher recurrence and mortality rates [3]. Early prediction of a pCR vs. a non-pCR to NAST can be helpful for clinical management. Patients predicted to have a non-pCR can be potentially triaged to alternative investigational therapies to minimize their exposure to the toxicities of an ineffective standard NAST. However, there is currently no reliable way of predicting in the clinic which patients undergoing NAST will have a pCR prior to or early during their NAST treatment.

Dynamic contrast-enhanced (DCE) MRI is a functional imaging modality that evaluates vascular and perfusional properties of tissue [4,5]. DCE MRI images can be analyzed semiquantitatively with parameter maps such as positive enhancement integral, signal enhancement ratio (SER), and maximum slope of increase. With proper modeling, DCE MRI can also be used to derive important pharmacokinetic tissue parameters, including blood volume, blood flow, and permeability constant [6,7,8,9,10,11,12]. These semiquantitative and quantitative parameters have been investigated both for diagnostic purposes and for predicting the treatment response of different types of cancers, including breast cancer. Investigators have assessed whether changes in tumor properties on MRI (e.g., tumor size, diffusion parameters, and tumoral heterogeneity) at an early stage of treatment are correlated with pCR [13,14,15,16,17,18].

Volumetric measurements on MRI, including tumor volume (TV), enhanced TV (ETV), and functional TV (FTV), have been reported to be useful biomarkers for prediction of breast cancer response to NAST [19,20,21,22]. TV is the ellipsoidal volume of a tumor calculated by multiplying the anteroposterior, craniocaudal, and transverse tumor dimensions. The ETV is the volume occupied by voxels that show enhancement at a specific time after contrast agent injection. The FTV is the subset of the ETV that corresponds to the enhanced voxels demonstrating percentage enhancement (PE) and SER above preset PE and SER thresholds [19]. By convention, PE is the change in signal intensity from a pre-contrast phase to an early post-contrast phase relative to the pre-contrast phase of the DCE image series. PE provides a measure of the signal enhancement due to the wash-in of contrast agent in tumors. SER is defined as the ratio of the change in signal intensity between the early post-contrast phase and the pre-contrast phase to the change in signal intensity between a late post-contrast phase and the pre-contrast phase [23]. SER is particularly helpful in identifying tissues with fast wash-out of contrast agent [24,25]. The FTV incorporates the active tumor regions that usually show rapid gain and rapid loss of enhancement on DCE MRI after the injection of contrast agent. Several studies have assessed the utility of FTV as a predictor of response to NAST using early-phase DCE images acquired at 1 min, 1.5 min, 2 min, and 2.5 min post-contrast agent injection after the first, third, and fourth cycles of NAST [13,21,22,26,27,28].

The objective of this study is to compare the performance of FTVs after 2 cycles and 4 cycles of therapy as predictors of response to NAST in patients with TNBC.

## 2. Materials and Methods

### 2.1. Patient Population

A total of 256 patients with stage I-III TNBC enrolled in a prospective clinical trial (NCT02276443) approved by the Institutional Review Board were considered for inclusion in the study. Written informed consent was obtained from all study participants. TNBC was defined from standard pathologic assays as negative for ER and PR (<10% tumor staining) and negative for HER2 (immunohistochemistry (IHC) score < 3, gene copy number not amplified). Patients with stage IV disease prior to the initiation of chemotherapy, or who have had a prior excisional biopsy of the primary invasive breast cancer, or who are not eligible for taxane and/or anthracycline-based chemotherapy regimens, were excluded from this study. Of the 256 patients, 154 did not have DCE MRI images at C2 and/or C4 and were thus excluded. Additionally, 1 patient was excluded due to a technical problem with the MR images and another patient was excluded due to the lack of a pathology report. Therefore, the study population consisted of the remaining 100 patients who received NAST and had MRI scans at baseline, after 2 cycles of NAST (C2), and after 4 cycles of NAST (C4). NAST consisted of dose-dense doxorubicin and cyclophosphamide-based chemotherapy for 4 cycles followed by paclitaxel every 2 weeks for 4 cycles or weekly for 12 doses. NAST was followed by surgery with a definitive pathological assessment of residual disease. pCR was defined as no residual invasive disease in the breast or in the resected axillary lymph nodes [21].

### 2.2. Image Acquisition

MRI scans were performed on a GE 3.0 T MR750w whole body scanner (Waukesha, WI) with a bilateral 8-channel phased array coil. The patients were placed in a prone and feet-in-first position for imaging. The imaging protocol included a T2-weighted series and a DCE MRI series based on differential subsampling with cartesian ordering (DISCO) sequence. Typical MRI scan parameters used for the DISCO acquisition were as follows: field of view = 34 × 34 cm, slice thickness = 3.0 mm, slice spacing = −1.5 mm, flip angle = 12°, repetition time = 7.6 ms, echo time 1/echo time 2 = 1.1/2.3 ms, total acquisition time = 7 min, matrix = 320 × 320, number of acquired slices = 60−115, in-plane spatial resolution = 0.6−0.8 mm, temporal resolution of DISCO series = 8−15.5 s, receiver bandwidth = ±166.7 kHz, and number of excitations = 0.69. At the start of the DCE MRI scan, a single bolus of gadobutrol (Gadovist, Bayer Health Care) contrast agent was injected (0.1 mL/kg at ~2 mL/second followed by saline flush) after obtaining at least one pre-contrast phase.

### 2.3. DCE MRI Analysis

**Figure 1:**
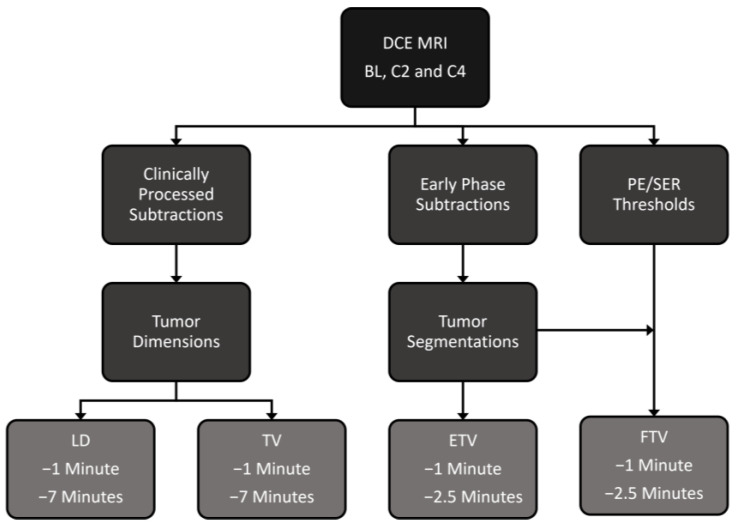
Flow chart shows the workflow of data acquisition, image processing, tumor segmentation, and measurement.

Pre-contrast, early (1 min and 2.5 min)-, and late (7 min)-phase DCE MRI images at BL, C2, and C4 were obtained using the imaging protocol and parameters described in Section 2.2. DCE subtraction images were obtained by subtracting the pre-contrast images from the early- and late-phase images. Figure 1 shows the workflow from acquisition of DCE-MR images to tumor segmentation and measurement.

Tumor dimensions along the anteroposterior, craniocaudal, and transverse directions were measured on DCE subtraction images acquired 1 min and 7 min after contrast agent injection, with the LD determined from these orthogonal measurements. TV at 1 min and then at 7 min were calculated as follows:

$$TV=\frac{4}{3}\pi \times \frac{Anteroposterior\;dimension}{2}\times \frac{Craniocaudal\;dimension}{2}\times \frac{Transverse\;dimension}{2}$$

**Figure 2:**
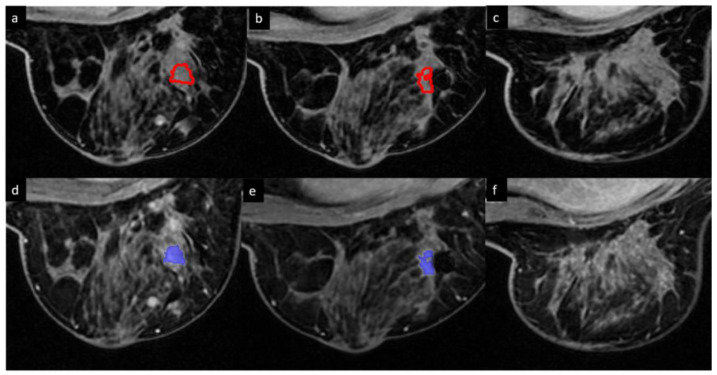
Enhanced tumor volumes (red) and functional tumor volumes (blue) in a 61-year-old patient with TNBC with a pathologic complete response at surgery. (**a**,**d**) Baseline. (**b**,**e**) After 2 cycles of neoadjuvant systemic therapy (NAST). (**c**,**f**) After 4 cycles of NAST. No segmentation was performed after 4 cycles since there was no residual tumor enhancement at that time. Functional tumor volume (**bottom row**) is the subset of enhanced tumor volume (**top row**), satisfying the PE and SER thresholds.

For ETV and FTV measurements, the phases at 1 min and 2.5 min after contrast agent injection were chosen as the early phases on the basis of previous studies by Musall et al. [28] and Hylton et al. [27], whereas the final phase of the DCE scan, at 7 min after contrast agent injection, was chosen as the late phase. Tumor contouring was performed by 2 breast fellowship-trained radiologists with 8 years and 4 years of experience, respectively, on the DCE subtraction images at baseline, C2, and C4 using an in-house image analysis software program (Image-I) [28]. Image-I offers a platform for the automated import/export of images and for manual as well as semiautomatic contouring of the regions of interest. The entire tumor volume from the 3D images was first manually segmented on a slice-by-slice basis, followed by semiautomatic refinement using the histogram thresholding feature of Image-I. Figure 2 shows ETVs and FTVs of a representative tumor at baseline, C2, and C4.

The ETVs at the different phases (e.g., 1 min and 2.5 min) were calculated as follows:

ETV=Voxel volume×Number of voxels in segmented region

For calculation of the FTV, the PE and the SER were defined as:

$$PE=\frac{S_{Early}-S_{Pre-contrast}}{S_{Pre-contrast}}\times 100$$

$$SER=\frac{S_{Early}-S_{Pre-contrast}}{S_{Late}-S_{Pre-contrast}}$$

where S_Early_, S_Pre-contrast_, and S_Late_ are the pixel intensity of the early-phase (1 min or 2.5 min after contrast agent injection), pre-contrast-phase (before contrast agent injection), and late-phase (7 min after contrast agent injection) images, respectively [29].

PE thresholds (from 0% to 220% in increments of 5%) and SER thresholds (from 0 to 2 in increments of 0.05) were predefined. For each combination of PE and SER thresholds, the number of ETV voxels with PE and SER above the threshold values was determined and multiplied by voxel volume to calculate FTV at 1 min and at 2.5 min:

FTV=Voxel volume×Number of voxels with PE and SER above threshold

For LD, TV, ETV, and FTV, relative changes between baseline and C2 (%C2/BL) were calculated as follows:

$$\%C2/\mathrm{BL}=\frac{C2-BL}{BL}\times 100$$

where C2 = measured values of LD, TV, ETV, or FTV after 2 cycles of NAST and BL = measured values of LD, TV, ETV, or FTV at baseline before NAST.

Similarly, relative changes between baseline and C4 (%C4/BL) were calculated for LD, TV, ETV, and FTV as follows:

$$\%C4/\mathrm{BL}=\frac{C4-BL}{BL}\times 100$$

where C4 = measured values of LD, TV, ETV, or FTV after 4 cycles of NAST and BL = measured values of LD, TV, ETV, or FTV at baseline before NAST.

### 2.4. Statistical Analysis

Volumetric measurements and LDs were compared between patients with pCR and patients with non-pCR by using the Mann–Whitney test and Fisher’s exact test. Analyses were conducted separately for baseline, C2, C4, %C2/BL, and %C4/BL measurements. For LD, TV, and ETV, area under the receiver operating characteristic curve (AUC) was measured to assess the performance in predicting pCR status. For FTV, 3D contour plots of the AUC by PE and SER were generated, and for each plot, the PE threshold and SER threshold corresponding to the maximum AUC were chosen as the optimal thresholds. For the cases with the maximum AUC corresponding to multiple PE and SER pairs, the pair with the minimum PE and SER values was chosen. FTV values corresponding to the optimal thresholds were then reported. The Mann–Whitney test was repeated to determine the statistical significance of the difference in the predictive performance of FTV at C2 and C4. *p*-values less than 0.05 were considered statistically significant. Statistical analysis was performed using R (version 4.0.3, R Development Core Team, Vienna, Austria).

## 3. Results

Patient characteristics are summarized in Table 1. All patients had their largest primary tumor diameter greater than 1.5 cm. The mean (standard deviation (SD)) largest primary tumor diameter was 3.4 cm (1.6 cm), and the median (range) largest primary tumor diameter was 2.8 cm (1.2−9.6 cm). Of the 100 patients included in the study, 51 (51%) had non-pCR and 49 (49%) had pCR.

LD, TV, and ETV measurements are summarized in Table 2 and FTV measurements are summarized in Table 3.

**Figure 3:**
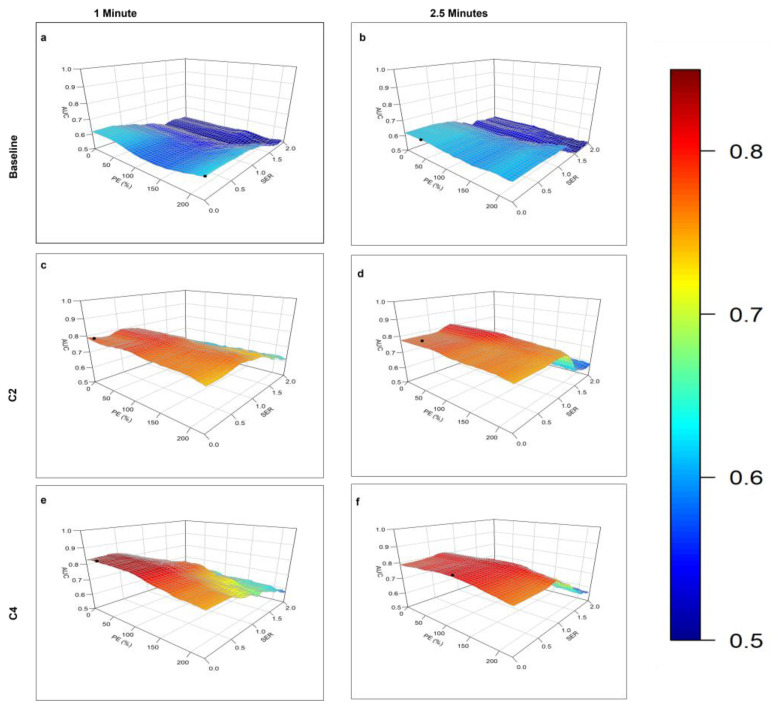
Plots of the area under the receiver operating characteristic curve (AUC) for response prediction as a function of the percentage enhancement (PE) and signal enhancement ratio (SER) used to calculate the functional tumor volume on DCE MRI images obtained (**a**,**b**) at baseline, 1 min (**a**), and 2.5 min (**b**) after contrast agent injection; (**c**,**d**) after 2 cycles of neoadjuvant systemic therapy (NAST), at 1 min (**c**), and 2.5 min (**d**) after contrast agent injection; and (**e**,**f**) after 4 cycles of NAST, at 1 min (**e**), and 2.5 min (**f**) after contrast agent injection. In each case, the PE–SER combination with maximum AUC (black circle) was selected as the optimal threshold.

**Figure 4:**
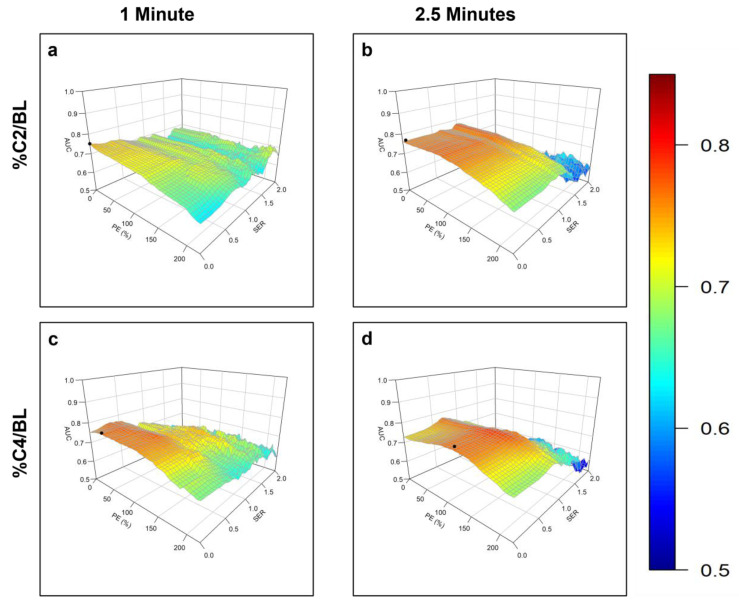
Plots of the area under the receiver operating characteristic curve (AUC) for response prediction as a function of the percentage enhancement (PE) and signal enhancement ratio (SER) used to calculate the functional tumor volume for relative changes between baseline and after 2 cycles of neoadjuvant systemic therapy (C2) (%C2/BL), and between baseline and after 4 cycles of neoadjuvant systemic therapy (C4) (%C4/BL): (**a**) %C2/BL per the early phase at 1 min, (**b**) %C2/BL per the early phase at 2.5 min, (**c**) %C4/BL per the early phase at 1 min, and (**d**) %C4/BL per the early phase at 2.5 min after contrast agent injection. In each case, the PE–SER combination with maximum AUC (black circle) was selected as the optimal threshold.

The contour plots of AUC vs. PE and SER for identification of the optimal PE and SER thresholds at different stages of NAST and for %C2/BL and %C4/BL are shown in Figure 3 and Figure 4.

At baseline, all AUC measurements for LD, TV, ETV, and FTV were less than 0.70 (*p* value > 0.001, 95% confidence interval with minimum *p* value of 0.029 for FTV at 1 min). Thus, none of the measured parameters at baseline were statistically significant predictors of pCR. At C2 and C4, all the measured parameters along with their changes relative to baseline had AUCs greater than 0.70 (*p* < 0.001).

The best AUC at C2 was achieved with FTV at 2.5 min at threshold PE and SER values of 60% and 0.90, respectively (AUC = 0.82, *p* < 0.001). The best AUC at C4 was achieved with FTV at 1 min with PE and SER thresholds of 30% and 0.40, respectively (AUC = 0.84, *p* < 0.001).

The ETV measurement at 1 min at C4 yielded the highest AUC of 0.82. Among all the measurements of LD, TV, ETV, and FTV, the highest AUC of 0.84 (*p* < 0.001) was achieved by FTV at 1 min at C4 with PE and SER thresholds of 30% and 0.40, respectively.

**Figure 5:**
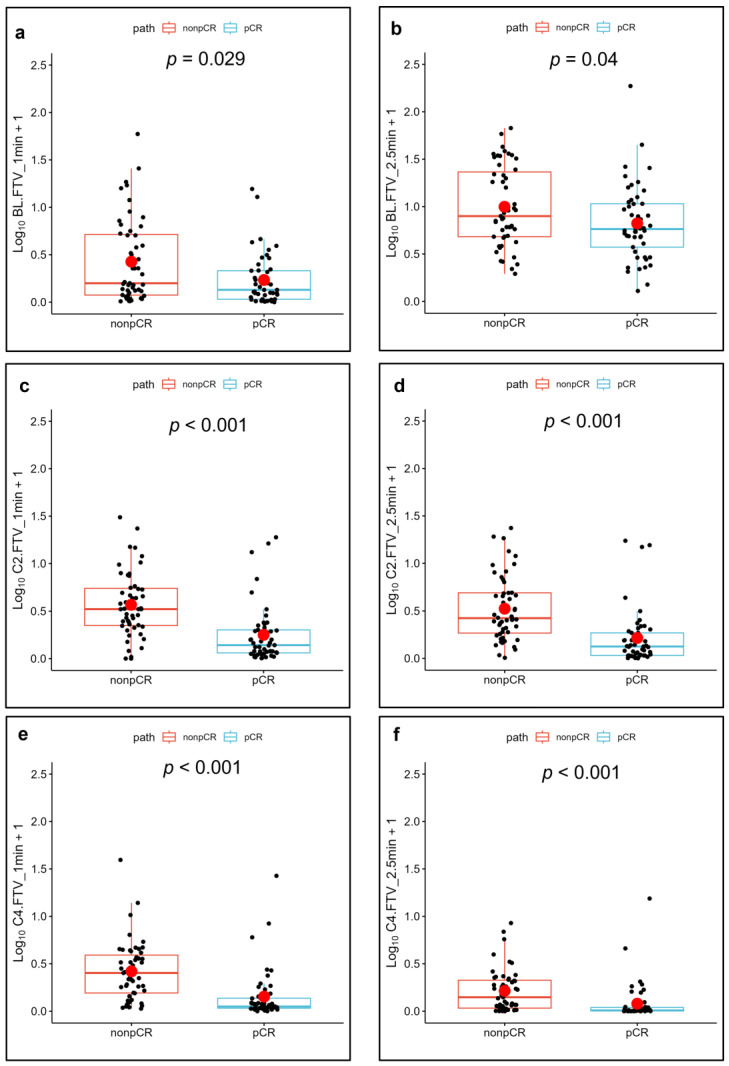
Functional tumor volume (FTV) plots and their mean (red circle) corresponding to optimal percentage enhancement (PE) and signal enhancement ratio (SER) for patients with pathologic complete response (pCR) and non-pCR. Plots correspond to images obtained (**a**,**b**) at baseline (BL), 1 min (**a**), and 2.5 min (**b**) after contrast agent injection; (**c**,**d**) after the second cycle of neoadjuvant systemic therapy (NAST) (C2), at 1 min (**c**), and 2.5 min (**d**) after contrast agent injection; and (**e**,**f**) after the fourth cycle of NAST (C4), at 1 min (**e**), and 2.5 min (**f**) after contrast agent injection.

**Figure 6:**
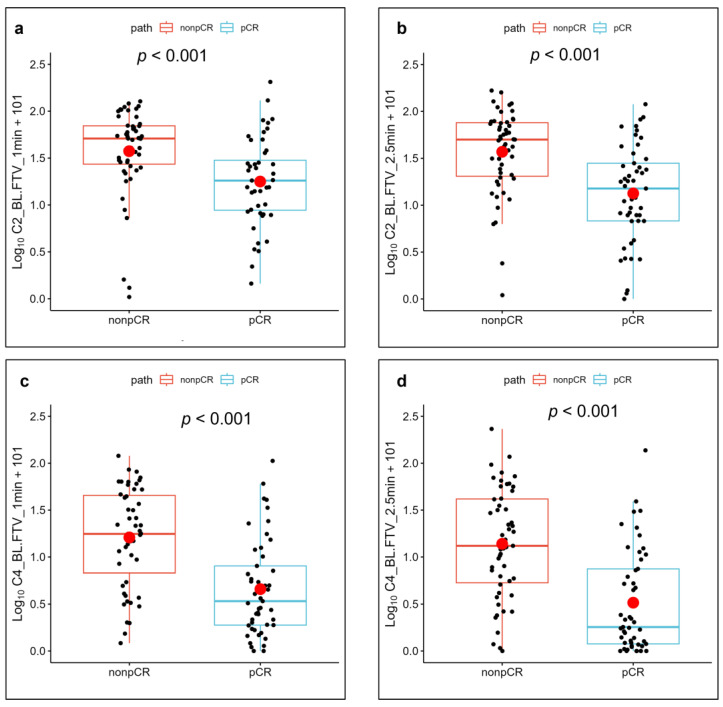
Relative changes in the functional tumor volume (FTV) measurements and their mean (red circle) corresponding to optimal percentage enhancement (PE) and signal enhancement ratio (SER) for patients with pathologic complete response (pCR) and non-pCR. Plots correspond to (**a**,**b**) changes in FTV from baseline (BL) to after the second cycle of neoadjuvant systemic therapy (NAST) (C2), measured on images obtained 1 min (**a**) and 2.5 min (**b**) after contrast agent injection; and (**c**,**d**) changes in FTV from baseline to after the fourth cycle of NAST (C4), measured on images obtained 1 min (**c**) and 2.5 min (**d**) after contrast agent injection.

Figure 5 and Figure 6 show the FTV measurements of the patients with a pCR and a non-pCR with their corresponding *p* values.

Even though the measured AUC values for LD and TV were slightly higher for 1 min than for 7 min (Table 2), the AUC values did not differ significantly between the 1 min and 7 min phases. Similarly, the AUC values for FTV did not differ significantly between the 1 min and 2.5 min phases (Table 3).

Comparisons of the predictive performances of LD, TV, ETV, and FTV at C2 and C4 by using the Mann–Whitney test revealed no significant differences in the pCR predictions between C2 and C4 (*p* > 0.001, 95% CI, minimum *p* value of 0.063 for TV at 7 min).

## 4. Discussion

In our study, FTVs based on longitudinal DCE MRI at C2 and C4 were found to successfully differentiate patients with pCR from those with non-pCR among TNBC patients undergoing NAST. This study demonstrates that all the measured parameters—LD, TV, ETV, and FTV—showed a good correlation with treatment response at C2 and C4 and for %C2/BL and %C4/BL. Among all the volume measurements (TV, ETV, and FTV), FTV exhibited higher values of AUCs at most time points. Furthermore, the predictive performance of the volume measurements at C2 was similar to their performance at C4. In comparison, the volume measurements and LDs measured at BL were unable to predict the treatment response. This study also showed that the early time phases 1 min and 2.5 min measurements were not statistically significantly different in their predictive performances.

The importance of early prediction of treatment response and the role of MRI in assessing tumoral characteristics before and during different stages of treatment are well known. ETV and FTV measurements have been demonstrated to be important biomarkers in the assessment of response to NAST and the prediction of recurrence-free survival [20,21,22,28,30]. However, we could find no published study comparing FTV after 2 cycles of NAST (early-on treatment) and after 4 cycles of NAST (mid treatment) as a treatment response predictor.

On the basis of the findings from the ACRIN 6657 trial in 230 patients with hormone receptor HR-positive/HER2-negative, HER2-positive, and HR-negative/HER2-negative (TNBC) breast cancer, Hylton et al. and Jafri et al. first reported FTV as an important biomarker in NAST treatment response assessment [20,21,30]. Li et al. showed the effect of imaging contrast thresholds in the prediction of breast cancer response to NAST [27]. For the 30 TNBC patients in their study, an AUC of 0.85 was reported for %C4/BL for FTV at 2.5 min with PE and SER threshold values of 140% and 0.0, respectively. In comparison, our study had a slightly lower AUC than that of Li et al. and showed a maximal AUC for %C4/BL for FTV at 2.5 min of 0.79 at threshold values of 125% and 0.70, respectively. Our study utilized a patient cohort approximately three times larger than that by Li et al., which could have contributed to the difference in AUCs observed in the results. Henderson et al. reported that the interim changes in ETV after 3 cycles of treatment exhibited better prediction of pathologic response than the interim changes in FTV [22]. Our study found that FTV and the relative changes in FTV showed a higher AUC than ETV and the relative changes in ETV for most of the measurements. However, the difference observed in ETV and FTV was not statistically significant. In addition to the differences in patient populations, the differences in the results may be due to the fact that Henderson et al. used a single PE of 50% in calculating FTV, whereas we established and used an optimal set of PE and SER thresholds to produce a maximum AUC. Our results showed different PE and SER thresholds corresponding to the maximum AUC for different timepoints and parameters. The exact cause for the variation of PE and SER threshold corresponding to the maximum AUC and its clinical significance are unclear and may require further investigation. One plausible contributing factor is the change in tumor vascularity at different time points from the neoadjuvant systemic treatment. Musall et al. performed a study in 60 TNBC patients to analyze the predictive performances of LD, TV, ETV, and FTV calculated using DCE MRI images acquired 1 min and 2.5 min after contrast agent injection at baseline and C4 [28]. The study reported FTV as the best performer among all the volume measurements, with an AUC of 0.85 (95% CI) for FTV at 1 min at C4. In addition, Musall et al. found that the early-phase timing of 1 min after contrast agent injection showed improved performance for predicting NAST response in TNBC in comparison to the early-phase timing of 2.5 min. There was an overlap of 17 (17%) study subjects between the patient population of Musall et al. and the patient population used in our study. Our results are in general agreement with the findings of the Musall study. However, we found that the FTV measurements at the early-phase timings of 1 min and 2.5 min after contrast agent injection had similar performance in predicting the response, even though the optimal thresholds of PE and SER were slightly different (Table 3). More importantly, our study revealed that DCE MRI acquired at C2 and DCE MRI acquired at C4 predicted the response with similar high performance (Table 3). Being able to predict patients’ response earlier in their treatment has the obvious advantage of enabling earlier changes in treatment, which may provide significant benefits to patients.

Our study has some limitations. First, the total size of our study population was limited which attenuated the power of the statistical analysis. We did, though, have 100 patients with TNBC who completed MRI at all three time points and underwent surgery with a pathologic confirmation of their treatment response. Second, our study was performed in a single institution and on a single MRI scanner platform. Therefore, our findings need to be validated for their robustness across different scanner platforms and in a multi-institution setting. Finally, the quantitative TV measurements relied on manual tumor segmentation by radiologists. The effect of inter-reader agreement in tumor segmentation and its impact on the predictive performances of LD, TV, ETV, and FTV was not investigated in this study.

Our study was limited to three time points—baseline, C2, and C4. The appreciable differences observed in the results between baseline and at C2 implies that it is not only the drug-induced mutation resistance, but rather the presence of a significant number of pre-existing cells resistant to the NAST, that influences the patient’s treatment response. Future work may include the analysis of images obtained after a single cycle of NAST.

## 5. Conclusions

FTV measurements from DCE MRI are useful biomarkers for discriminating TNBC patients with pCR and non-pCR to NAST. In particular, FTVs by DCE MRI after 2 cycles of treatment are able to predict the treatment response with high performance similar to that of FTVs after 4 cycles of treatment, thus providing an earlier opportunity for modifying the patient’s treatment course if warranted. Furthermore, both early phases—1 min and 2.5 min—showed similar predictive performances.

## Figures and Tables

**Table 1:** Characteristics of the study patients with triple-negative breast cancer who received neoadjuvant systemic therapy.

Characteristic	Value			*p*-Value
Age, years	Total (N = 100)	pCR (N = 49)	Non-pCR (N = 51)	0.893
Mean (SD)	47.9 (10.8)	47.5 (10.5)	48.3 (11.2)	
Median (range)	48 (23–77)	48 (23–66)	48 (31–77)	
Longest tumor diameter, cm				
Mean (SD)	3.4 (1.6)	3.2 (1.8)	3.6 (1.5)	N/A
Median (range)	2.8 (1.2–9.6)	2.7 (1.2–9.6)	2.9 (1.6–7.9)	
Histologic type, n (%)				0.503
Invasive ductal	87 (87)	45 (92)	42 (82)	
Metaplastic	10 (10)	3 (6)	7 (14)	
Poorly differentiated carcinoma	1 (1)	1 (2)	0	
Apocrine	1 (1)	0	1 (2)	
Invasive mixed ductal/lobular	1 (1)	0	1 (2)	
T category, n (%)				0.130
T1	15 (15)	11 (23)	4 (8)	
T2	63 (63)	30 (61)	33 (65)	
T3	16 (16)	6 (12)	10 (19)	
T4	6 (6)	2 (4)	4 (8)	
N category, n (%)				0.052
N0	64 (64)	36 (74)	28 (55)	
N1	20 (20)	6 (12)	14 (27)	
N2	4 (4)	1 (2)	2 (4)	
N3	12 (12)	6 (12)	7 (14)	
Overall clinical stage, n (%)				0.141
I	10 (10)	7 (14)	3 (6)	
II	68 (68)	34 (70)	34 (67)	
III	22 (22)	8 (16)	14 (27)	
Type of surgery, n (%)				N/A
Total mastectomy	45 (45)	21 (43)	24 (47)	
Breast-conserving surgery	55 (55)	28 (57)	27 (53)	

SD, standard deviation; pCR, pathologic complete response.

**Table 2:** Longest tumor dimension (LD), tumor volume (TV), and enhanced tumor volume (ETV) measurements by neoadjuvant systemic therapy response status.

Measurement	AUC [95% CI]	*p*-Value	Mean ± SD		Best Cutoff for ROC Curve
			Non-pCR (N = 51)	pCR (N = 49)	
LD at 1 min, cm					
BL	0.58 [0.47–0.70]	0.148	3.58 ± 1.54	3.24 ± 1.75	4.35
C2	0.72 [0.61–0.82]	<0.001	2.92 ± 1.45	2.06 ± 1.34	1.45
C4	0.76 [0.66–0.85]	<0.001	2.34 ± 1.41	1.74 ± 1.57	0.95
%C2/BL	0.75 [0.66–0.85]	<0.001	−16.94 ± 21.88	−36.12 ± 22.10	−19.38
%C4/BL	0.77 [0.68–0.86]	<0.001	−32.58 ± 27.78	−67.88 ± 34.78	−49.58
LD at 7 min, cm					
BL	0.58 [0.47–0.70]	0.156	3.71 ± 1.51	3.39 ± 1.77	4.20
C2	0.71 [0.61–0.81]	<0.001	3.11 ± 1.49	2.25 ± 1.33	2.45
C4	0.76 [0.66–0.85]	<0.001	2.71 ± 1.72	1.39 ± 1.52	1.35
%C2/BL	0.76 [0.67–0.86]	<0.001	−14.84 ± 21.46	−31.24 ± 18.37	−15.39
%C4/BL	0.75 [0.65–0.84]	<0.001	−23.91 ± 47.09	−58.85 ± 35.06	−49.14
TV at 1 min, cm^3^					
BL	0.61 [0.50–0.72]	0.061	24.22 ± 32.09	15.14 ± 27.26	22.65
C2	0.73 [0.63–0.83]	<0.001	12.80 ± 18.53	4.72 ± 8.02	4.42
C4	0.77 [0.68–0.86]	<0.001	7.55 ± 15.63	2.20 ± 5.47	0.32
%C2/BL	0.77 [0.67–0.86]	<0.001	−39.21 ± 39.23	−69.29 ± 26.64	−67.46
%C4/BL	0.77 [0.68–0.87]	<0.001	−62.41 ± 39.34	−86.94 ± 23.91	−99.40
TV at 7 min, cm^3^					
BL	0.60 [0.49–0.71]	0.080	25.23 ± 32.61	16.62 ± 28.24	30.37
C2	0.73 [0.63–0.83]	<0.001	13.65 ± 19.84	5.53 ± 8.98	4.21
C4	0.77 [0.67–0.86]	<0.001	8.84 ± 17.45	2.69 ± 6.35	0.77
%C2/BL	0.75 [0.65–0.85]	<0.001	−38.51 ± 35.95	−66.41 ± 22.50	−56.42
%C4/BL	0.76 [0.67–0.85]	<0.001	−59.77 ± 40.96	−85.73 ± 19.25	−72.83
ETV at 1 min, cm^3^					
BL	0.62 [0.51–0.73]	0.038	12.98 ± 13.89	10.14 ± 23.64	13.55
C2	0.80 [0.70–0.89]	<0.001	5.30 ± 6.07	2.09 ± 4.19	1.25
C4	0.82 [0.73–0.90]	<0.001	2.75 ± 5.58	1.07 ± 3.82	0.54
%C2/BL	0.76 [0.66–0.86]	<0.001	−45.60 ± 31.91	−72.06 ± 26.00	−69.14
%C4/BL	0.73 [0.62–0.83]	<0.001	−71.15 ± 27.17	−86.06 ± 21.81	−87.58
ETV at 2.5 min, cm^3^					
BL	0.62 [0.51–0.73]	0.038	14.70 ± 15.44	11.20 ± 26.82	17.18
C2	0.78 [0.68–0.87]	<0.001	6.26 ± 6.84	2.54 ± 4.59	1.99
C4	0.79 [0.70–0.88]	<0.001	3.40 ± 6.73	1.13 ± 3.80	0.39
%C2/BL	0.74 [0.64–0.84]	<0.001	−43.25 ± 35.44	−69.60 ± 27.30	−63.88
%C4/BL	0.72 [0.62–0.82]	<0.001	−69.12 ± 31.05	−87.36 ± 18.42	−88.28

AUC, area under the receiver operating characteristic curve; SD, standard deviation; pCR, pathologic complete response; BL, baseline; C2, after 2 cycles of neoadjuvant systemic therapy (NAST); C4, after 4 cycles of NAST; %C2/BL, change between baseline and C2; %C4/BL, change between baseline and C4.

**Table 3:** Functional tumor volume (FTV) measurements by neoadjuvant systemic therapy response status.

Measurement	Optimal Threshold		AUC [95% CI]	*p*-Value	Mean ± SD (cm^3^)		Best Cutoff for ROC Curve
	PE (%)	SER			Non-pCR (N = 51)	pCR (N = 49)	
FTV at 1 min							
BL	220	0.25	0.63 [0.52−0.74]	0.029	4.22 ± 9.30	1.25 ± 2.67	3.84
C2	20	0.55	0.80 [0.70−0.89]	<0.001	4.22 ± 5.58	1.60 ± 3.69	1.40
C4	30	0.40	0.84 [0.76−0.92]	<0.001	2.74 ± 5.58	1.05 ± 3.82	0.12
%C2/BL	0	0	0.75 [0.65−0.85]	<0.001	−48.01 ± 34.09	−64.94 ± 55.90	−71.19
%C4/BL	35	0.35	0.78 [0.68−0.87]	<0.001	−72.64 ± 27.11	−90.56 ± 18.59	−92.75
FTV at 2.5 min							
BL	0	0.30	0.62 [0.51−0.73]	0.040	14.66 ± 15.43	11.16 ± 26.67	17.18
C2	60	0.90	0.82 [0.73−0.90]	<0.001	3.76 ± 4.89	1.39 ± 3.55	0.56
C4	125	0.75	0.82 [0.73−0.91]	<0.001	0.93 ± 1.48	0.49 ± 2.10	0.09
%C2/BL	0	1.00	0.78 [0.68−0.87]	<0.001	−47.06 ± 39.97	−76.96 ± 26.16	−86.11
%C4/BL	125	0.70	0.79 [0.70−0.88]	<0.001	−71.15 ± 39.99	−92.07 ± 20.65	−98.48

PE, percentage enhancement; SER, signal enhancement ratio; SD, standard deviation; pCR, pathologic complete response; BL, baseline; C2, after 2 cycles of neoadjuvant systemic therapy (NAST); C4, after 4 cycles of NAST; %C2/BL, change between baseline and C2; %C4/BL, change between baseline and C4.

## Data Availability

Data are available from the corresponding author upon request.
